# Developing a Stigma Scale for the Workplace: Focus on an Airline Cabin Crew

**DOI:** 10.3390/ijerph18084003

**Published:** 2021-04-11

**Authors:** Haeok Liz Kim, Sunghyup Sean Hyun

**Affiliations:** 1 Computational Social Science Center, Hanyang University, Seoul 04763, Korea; lette0704@hanyang.ac.kr; 2 School of Tourism, Hanyang University, Seoul 04763, Korea

**Keywords:** stigma, stigma scale, criterion validity, scale development, airline cabin crew

## Abstract

The purpose of this study is to develop metrics for stigma-producing factors by conducting a survey among stigma inflictors, that is, people who participate in the stigmatization of others. This study attempted to develop a stigma measurement scale for service industry workers. This study focused airline cabin crew members in accordance with a seven-step scale development procedure to derive stigma measurement variables. As a result, the stigma scale developed in this study consists of 6 factors (work ability, conscientiousness, selfishness, work ethics, appearance, neuroticism) containing 28 measurement items. This study indicates the need to find countermeasures that can reduce stigmatization of employees within organizations. At a personal level, the practical implication is to prevent stigmatization from occurring within the organization by improving the perception of stigma by cabin crew within the airline organization. At the organizational level, the practical implication is to analyze and reduce the factors of social stigma that negatively affect organizational performance.

## 1. Introduction

Human prejudices greatly influence decision making [1,2]. First impressions and stereotypes can affect social interactions leading to behavioral confirmation, making false impressions look like reality [3,4,5,6]. Prejudices or stereotypes can persist for a long time causing a positive or negative attitude toward a subject [1].

In most social environments, we interact with various people. When we perceive negative attributes in strangers, we often treat them with disdain and value them less than other ordinary people who are aligned with social norms. The phenomenon where a person is perceived as less worthy of honor, due to a certain attribute, by almost the entire society is defined by scholars as stigma [7].

Social stigma is the damaged social identity of a person [7]. It refers to the social alienation, widespread disapproval, and discrimination imposed on a person due to their having certain attributes that make them different or abnormal [7,8]. Social stigma is a multi-dimensional concept that includes stereotypes, discrimination, and prejudice about certain people who become stigmatized by others [9].

Most research on stigma consists of medical and psychosocial studies on the effects of stigmas caused by acquired immune deficiency syndrome (AIDS), mental illness, alcoholism, physical disability, and smoking, etc. The areas of interest were mainly mental and physical damages caused by stigma, and education, treatment, and countermeasures to solve them. Recently, the research field has been expanding to include stigmas on brands and corporate bankruptcy, the impact of stigma on organizational members, and stigmas that occur within an organization. However, there has been little research on stigmas on workers in the hospitality industry. This study attempts to develop a stigma scale by identifying factors that cause airline cabin crew workers to be stigmatized by their co-workers.

Considering the hierarchical team-based structure of cabin crews, when stigma occurs in the organization, the victims are mostly juniors, not seniors. This study analyzed the stigma phenomenon in airline organizations from the perspective of the inflictors (seniors who impose stigmas on juniors) and developed a measure of social stigma. The development of a stigma scale for airline cabin crew may contribute to preventing unnecessary conflicts within the organization by helping us to understand the stigma phenomenon in the service industry and identifying factors that cause stigma within cabin crews.

This study highlights the need to form countermeasures for reducing stigma within the organization. At a personal level, the practical implication is to prevent stigma from occurring within the organization by improving the perception of stigma by a cabin crew within an airline organization. At the organizational level, the practical implication is to analyze and reduce the factors of social stigma that negatively affect organizational performance.

## 2. Conceptual Framework

### 2.1. Stigma

Stigma is a phenomenon in which an individual possesses a degraded social identity due to a group’s prejudice towards that individual within a social context [10]. Stafford and Scott (1986) defined stigma as a characteristic possessed by a person that is contrary to social norms [11]. Stigma refers to the damaged social identity, social alienation, and negative treatment suffered by a person due to their attributes that are perceived as different, unacceptable, and undesirable by others [7,8].

The term stigma relates back to the ancient Greeks, who burned or cut marks into the skin of slaves, criminals, and traitors in order to identify them as immoral or tainted people who should be avoided [7]. Today, stigma is no longer a physical mark but it is still associated with widespread social disapproval—a discrediting social difference that yields a “spoiled social identity”, to use Goffman’s terms [11]. Goffman (1963) is a leading scholar who studied stigma in social psychology. In his book *Stigma: notes on the management of spoiled identity*, basic concepts and approaches were classified from the perspective of social psychology. The three distinctions for stigma are ethnic identity, personal character flaws, and abomination of the body. Link and Phelan (2001) have suggested a multidimensional model in which the concept of stigma is separated into various components, including: discrimination, stereotyping, labelling, separation, and status loss within the context of power differential [12].

### 2.2. Measurement Items for Criterion Validity

Newly developed measures or scales should have an empirical connection to “gold standard”-like standards. This is known as criterion validity [13], and means that one measure is related to other measures [14]. Criterion validity represents the statistical equivalence between the estimated and known parameters [15].

### 2.3. Stigma Criterion Validity for Airline Cabin Crew

Job Competency (JC)

Job competency refers to an individual’s characteristics and abilities that are exhibited when performing a job [16]. Competency generally refers to an individual attribute which is inherent to a person’s actions [17,18]. Previous studies have explained that competency has two distinct elements: acquired skills and innate traits [17]. Competency includes inherent human characteristics, such as motivation, self-image, effort, values, enthusiasm, standards, and moral criteria [19].

Work-related competency of the cabin crew is crucial to an airline company because success in the airline industry depends largely on the quality of service provided by the cabin crew [19]. Also, the cabin crew should ensure that all aspects of security and safety duties are completed during the flight. In order to perform these duties, cabin crew must comply with the aviation safety procedure and company policy, as well as update safety information before flight. This is the why airlines strive to employ well-qualified employees. If the cabin crew’s job competency is insufficient, teamwork and team performance are adversely affected.

Conscientiousness (CS)

McCrae and Costa (1985) developed NEO (NEO-PI: Personality Inventory), a personality test that measures the reliability and validity of the Big Five [20]. As per NEO-PI, conscientious individuals are naturally well-organized, neat, thorough, able to hold their impulses in check, meticulous, persistent, dependable, industrious, trustworthy, and goal-driven go-getters [21,22,23,24]. Conscientious people are discerning and prudent, perform their duties responsibly, have excellent organizational skills, and have a strong desire for achievement [21].

Researchers have found that conscientious employees: are not only more punctual [22,25], but also tend to outperform their less conscientious coworkers [26,27]; have more positive attitudes toward theirs plans [28]; and work more diligently than their non-conscientious coworkers [29,30]. Conscientious employees are likely to spend more time working [31], put more effort into their work [32], have more job-related knowledge, and achieve a higher quantity and quality of work. All these factors together create a positive effect on job performance. Therefore, conscientiousness in individual cabin crew members is critical for achieving the team’s performance.

Honesty (HH)

The HEXACO( model divides personality into six dimensions: honesty–humility, emotionality, extraversion, agreeableness, conscientiousness, and openness to experience [33]. Honesty–humility is defined on its positive pole by fairness, sincerity, modesty, and greed avoidance, and on its negative pole by greediness, slyness, injustice, and pretentiousness [34].

At the workplace, the negative pole of honesty–humility has been associated with workplace delinquency or anti-social behavior [35,36], counterproductive work behavior [37,38], unethical leadership [39], and sexual harassment [40]. People low in honesty–humility seem to have a greater tendency to abuse their power or privileges granted by the organization for personal–e.g., material, social, and sexual—gains [34].

Such behavior can be a direct cause of stigma within an organization. Stigma’s early perceptions of moral elements stem from the thoughts of the most common people, and this moral experience refers to a record through practical practice that defines the most important things in everyday life [7,41,42,43,44]. The relationship between moral experience and stigma shapes the stigma process of becoming an inflictor and a victim [44].

Leader Member Exchange (LMX)

Leader member exchange (LMX) is defined as the exchange relationship with the leader from a subordinate’s point of view. Leaders and subordinates affect each other’s work by forming a social exchange relationship [45]. LMX theory was created to describe the quality of the exchange relationship between leaders and team or organization members [46,47,48]. It refers to creating an environment based on values promoting interdependent behavior patterns in the team members’ mutual relationships in order to promote a feeling of shared consequences [49]. In the interaction stage, the leader and members each have different physical characteristics, attitudes, backgrounds, abilities, personalities, and dispositions, which are factors that influence personal interaction [46].

The organization of airline cabin crew, which is a representative service organization, has introduced a team system to improve service quality and efficiency of manpower management through teamwork.

Physical Attractiveness (PA)

Physical appearance is characterized by a frontline employee’s facial features, physique, and attire [50,51,52]. Söderlund and Julander (2009) argued that physical attractiveness is an evaluation dimension used to evaluate individuals visually, immediately, and automatically [53]. It was found that physical attractiveness is used by people as a criterion while evaluating others [54,55]. The physical attractiveness stereotype is a concept that explains the positive and negative reactions of people to physically attractive individuals [56]. Physical attractiveness can be objectively measured by physical characteristics such as facial symmetry, height, skin color, and eye color [56,57,58].

Many studies have found that attractive physical appearance of frontline service providers helps to create positive impressions on customers [50,52]. Physically attractive employees are perceived as more persuasive, warmer, and friendlier than unattractive ones [51,59,60,61]. For this reason, not only airlines, but flight attendants themselves always pay attention to grooming in order to maintain an attractive appearance at work.

Mental Health (MH)

In response to the economic and social impacts of common mental health disorders such as depression [62], a combined approach to improving mental health in the workplace has been supported [63,64]. Prevention of mental health disorders via modification or elimination of work-related psychosocial risk constituents is key to promoting mental health [65].

Positive mental health is expressed as psychological well-being, and negative mental health is expressed as psychological stress such as depression, anxiety, and loss of emotional and behavioral control [65]. The stigma of mental health appears in the process of objectifying and depersonalizing a person classified as mentally ill [66]. Mental health stigma is a social cognitive process in which the public recognizes certain clues about an individual’s mental health status, which activates stereotypes and can lead to prejudice and discrimination [8,67,68,69]. However, there are few cases of controlled studies that evaluate the interventions impact of mental health literacy (MHL) on stigmatizing attitudes that are related to the workplace [64,70].

Psychological Well-Being (PW)

Psychological well-being is similar to the concept of welfare or happiness, but it is hard to define clearly because the meaning varies according to time, people, and place [71]. Psychological well-being refers to a participatory life in which an individual seeks the true meaning of life, possesses the ability to cope with difficulties, and strives to meet their inner beliefs [72,73].

Psychological well-being indicates accepting oneself positively, forming relationships with people, performing autonomous actions, and taking independent decisions in accordance with the standards and principles set by oneself [74]. Psychological well-being of a service provider not only affects his or her own life but also affects sales performance, customer attitude, and customer satisfaction [71]. An employee with psychological well-being is more likely to help or benefit others [75]. Also, psychological well-being fosters respect and trust among co-workers and positive relationships with superiors and other members in the organization [76]. Being stigmatized has detrimental consequences on the psychological well-being of a person [77,78].

Perceived Insider Status (PIS)

Perceived insider status refers to the degree to which an employee feels like an insider at the workplace [79]. It describes the sense of belonging felt by individuals in differentiated categories within the organization [80]. Perceived insider status can also be an important tool in the process of organizational action to produce positive results in an insider position compared to outsiders [81].

Employees who have a high insider status are more likely to have positive organizational attitudes (e.g., job satisfaction: [81]; affective commitment: [82]; intention to stay: [83]) and more likely to behave in ways helping organizational functions (e.g., higher task performance and innovative behavior: [81]). In order to distinguish it from the general outsider perspective, belonging is described as feeling like an insider when perceiving the reactions, experiences, and beliefs of one’s group regarding stereotypes or stigma [84].

## 3. Methods

### 3.1. Participants

Study 1: The first questionnaire survey (pre-test) was conducted to verify whether the initial items derived through expert surveys were suitable for the cabin crew stigma evaluation scale. The data collection period was for about two weeks from 10 September to 22 September 2020 and the data were collected at Incheon Airport and Gimpo Airport in South Korea. A total of 174 questionnaires were collected from Korean cabin crew members working at domestic airlines, out of which 4 were excluded due to reliability concerns and 170 were used in the analysis. To understand the demographic characteristics of this study, a total of 170 questionnaires were analyzed using the SPSS 24 statistical program.

Study 2: The main survey was conducted to verify whether the refined items through the 170 questionnaires were suitable for the cabin crew stigma evaluation scale. A total of 29 questions were derived from the results of the exploratory factor analysis conducted through the first pre-survey, and the questionnaire was modified and distributed. The main survey data collection lasted for 16 days from 25 September to 10 October 2020, and was conducted at Gimpo Airport in South Korea with Korean cabin crews working for domestic airlines. A total of 179 questionnaires were collected and 172 were used for the analysis, excluding seven unreliable ones. The 172 questionnaires were analyzed using the SPSS 24 statistical program to analyze the demographic characteristics of the survey.

Study 3: A total of 353 questionnaires were collected based on the pre survey and the main survey, and 342 were used for analysis, excluding 11 unreliable ones. In order to understand the demographic characteristics of this survey, the 342 questionnaires were analyzed using the SPSS/AMOS 24 statistical program. The frequency analysis results consisted of gender, position, marital status, education level, age, annual salary, and job tenure (number of years worked).

### 3.2. Instruments

**Figure 1:**
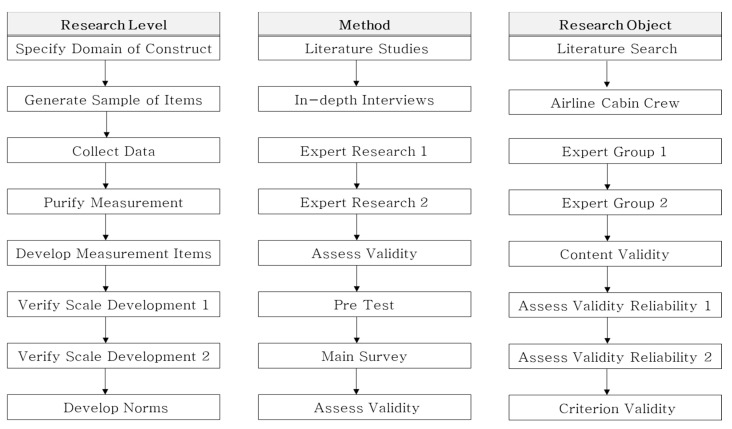
Research procedure.

In this study, we attempted to develop a scale based on previous studies of [14,85,86,87,88,89], who performed scale development. The scale development procedure of this study is showed in Figure 1.

First, in the research stage, related literature was investigated for the composition of initial items. This literature review was conducted in order to use in-depth interviews conducted for scale development for reference validation analysis to find out the correlation between refined measurement items and refined measurement variables based on literature research. Second, initial items were derived by conducting in-depth interviews with airline cabin crew members. Based on the extracted contents, the measurement items were refined through the process of correction, deletion, and addition through the opinions of experts. Third, based on the in-depth interviews, key words were selected and the first expert survey was conducted on a total of 10 people, consisting of academia and field experts. Reflecting the opinions of experts, measurement items were refined through deletion, modification, and addition. Fourth, the second expert survey was conducted based on the measured items refined through the first expert survey. The procedure was the same as that of the first item refinement. Fifth, the measurement items were developed through content validity verification. The content validity ratio (CVR) and a 5-point Likert scale average are calculated to verify the validity of the content, and statistically insignificant values were removed from the items. Sixth, a study 1 (pre-test) was conducted to verify the first scale, and validity factor analysis and confirmatory factor analysis were conducted based on the collected questionnaire data to verify validity and reliability.

This survey was conducted to perform the 2nd scale verification by supplementing and revising the 1st scale verification step. To evaluate the constructed scale, the dimensionality was re-verified through confirmatory factor analysis and the construction validity was verified. Finally, statistical significance was reviewed through the criterion validity to see if the airline cabin crew stigma factors derived through previous studies and in-depth interviews are suitable and have any correlation. For the criterion validity analysis, Pearson correlation analysis was performed to verify the statistical significance between variables.

### 3.3. Data Collection Process

This study was conducted on airline cabin crew members in accordance with a seven-step scale development procedure to derive stigma measurement variables. Through a literature review, a total of eight stigma factors (job competency, conscientiousness, honesty-humility, leader member exchange, physical attractiveness, mental health, psychological well-being, perceived insider status) and initial items were derived. Based on previous studies, in-depth interviews were conducted with cabin crew members using free association questionnaires and the validity of the content was reviewed by referring to expert opinions. Based on this, the first and second expert surveys were conducted to refine the airline cabin crew stigma factors into seven factors (work ability, conscientiousness, appearance, work ethics, selfishness, neuroticism, and human relations) and 41 initial items.

Study 1 was conducted on 170 airline cabin crew members through a questionnaire consisting of the derived measurement items. As a result of conducting exploratory factor analysis (EFA) and confirmatory factor analysis (CFA), 7 factors and 41 items were reduced to 6 factors and 29 items, deleting 12 items. To verify that the items refined in the first pre-test were suitable for the cabin crew stigma evaluation scale, a second survey was conducted on airline cabin crew members. Confirmatory factor analysis was conducted using 172 questionnaires, and one factor was deleted because it did not show statistical significance, and a total of 6 factors (work ability, conscientiousness, selfishness, work ethics, appearance, neuroticism) and 28 measurement items were derived. Finally, to verify the theoretical validity of the airline cabin crew stigma, based on the 342 questionnaires collected in the first and second surveys, criterion validity analysis was conducted on 8 leading factors presented through literature study and the 6 factors derived from the in-depth interviews. As a result of Pearson correlation analysis, it was found that the correlations between all variables were statistically significant.

### 3.4. Data Analysis

In the research analysis, we utilized program of IBM SPSS Statistics 21 and AMOS 21 for all statistical analyses. Descriptive statistics were utilized to identify the sample characteristics. To achieve research objectives, we followed Churchill [85]; DeVellis [13]; Hinkin, Tracey and Enz [88]; and the seven-step procedure of Clemenz [87] involving the measurement and structural model assessments. Specifically, we tested by employing exploratory factor analysis (EFA) and a confirmatory factory analysis (CFA) for data quality comprising construct validity and composite reliability.

## 4. Results

### 4.1. Data Quality Assessment

Step 1: Based on literature studies, in-depth interviews were conducted with airline cabin crew. The following specific questions were asked.

1. Have you ever had a stigma in your workplace?
2. Have you ever experienced being stigmatized in your workplace while working at an airline?
3. Have you ever seen someone else experience stigma? (e.g., co-workers or juniors being stigmatized due to insincere work, poor appearance, being late for briefings, frequent sick leave, etc.)

Step 2: This study refined the measurement items based on literature studies, in-depth interviews, and first and second expert surveys. As shown in Table 1 below, the scale was composed of 7 dimensions and 41 items.

### 4.2. Results of Demographic Profile

Study 1: Among the 170 survey participants, 145 (85.5%) were female and 25 (14.7%) were male. The participants were asked to indicate their annual incomes. Most respondents had an annual income of 30,000,000 to 39,999,999 Korean won (31.8%), followed by 40,000,000 to 49,999,999 won (27.1%), and 50,000,000 to 59,000,000 (23.2%). Regarding education level, 123 (72.4%) had a bachelor’s degree, followed by 26 (15.3%) graduate-degree holders, 20 (11.8%) 2-year college or some college graduates, and 1 (0.6%) high-school graduate or less. The survey also asked participants their work tenure. The majority (47, 27.6%) replied more than 10 years. As for respondents’ age, the age group with the highest proportion (82, 48.2%) was 30–39 years, followed by 20–29 years (72, 42.2%), and 40–49 years (16, 9.4%). Regarding job positions, most of them, that is, 86 (50.6%), were flight attendants (SS/SD), 40 (23.5%) were assistant pursers (AP), 35 (20.6%) were pursers (PS), 8 (4.7%) were senior pursers (SP), and 1 (0.6%) was chief purser (CP).

Study 2: Among the 172 survey participants, 152 (88.4) % were female, and 20 (11.6) % were male. The majority reported an income of 30,000,000 to 39,999,999 Korean won (72, 41.9%), followed by 40,000,000 to 49,999,999 won (51, 29.7%), and 50,000,000 to 59,000,000 (24, 14.0%). Regarding education level, 124 (72.1%) respondents had a bachelor’s degree, followed by 31 (18.0%) college graduates or 2-year college degree holders, and 17 (9.9%) graduate-degree holders. The survey also asked participants their work tenure. The majority (54, 31.4%) had been working for 5–7 years. The majority age group (86, 50.0%) was 20–29 years, followed by 30–39 years (65, 37.8%), and 40–49 years (21, 12.2%). Regarding job positions, 99 (57.6%) were flight attendants (SS/SD), 45 (26.2%) were assistant pursers (AP), 20 (11.6%) were pursers (PS), 7 (4.1%) were senior pursers (SP), and 1 (0.6%) was chief purser (CP).

Study 3: Among the 342 (170 + 172) survey respondents, 297 (86.8%) were female, and 45 (13.2%) were male. The most common annual income was 30,000,000 to 39,999,999 Korean won (126, 36.8%), followed by 40,000,000 to 49,999,999 Korean won (97, 28.4%), 50,000,000 to 59,999,999 Korean won (65, 19.0%), 60,000,000 won or more (42, 12.3%), and 24,999,999 won or less (12, 3.5%). Regarding education level, 247 (72.2%) respondents had a bachelor’s degree, followed by 51 (14.9%) 2-year college or some college graduates, 43 (12.6%) graduate-degree holders, and 1 (0.3%) respondent with a high-school graduate degree or less. The majority were of age 20–29 years (158, 46.2%) followed by 30–39 years (157, 43.0%) and 40–49 years (37, 10.8%). Regarding the job positions, 185 (54.1%) were flight attendants (SS/SD), 85 (24.9%) were assistant pursers (AP), 55 (16.1%) were pursers (PS), 15 (4.4%) were senior pursers (SP), and 2 (0.6%) were chief pursers (CP).

### 4.3. Data Quality Assessment

#### 4.3.1. Study 1: Exploratory Factor Analysis (EFA) vs. Confirmatory Factor Analysis (CFA)

As a result of conducting exploratory factor analysis in the initial items, a total of 20 items were deleted from the initial items reducing 8 factors (49 items) to 6 factors (29 items). In addition, neuroticism and human relations were grouped into the same factor, forming a single factor. (Table 2) shows the results of exploratory factor analysis refined from the initial items.

As shown in Table 2, the Kaiser–Meyer–Olkin (KMO) value shows a high value of 0.8 or higher, and Bartlett’s sphericality test is also suitable with a significant probability of 0.000 (*χ*^2^ = 3568.284, *df* = 406, *p* < 0.001). The total cumulative proportion of variance is 71.931%, which satisfies the appropriate level of 70% or more, and all factor loadings are 0.6 or more, suggesting that the convergent validity of the items has been secured.

Cronbach’s alpha(α) reflects the internal consistency reliability among indicators of a construct [90]. With values ranging from 0 to 1.0, the higher values indicate a higher reliability among the items. As shown in Table 2, The overall Cronbach’s alpha scores were high at 0.8.

Commonality of 0.4 or less consisted of a total of 29 items excluding 12. The 12 items which were deleted included three items of work ability (work ability 6, 7, 8), one selfishness item (selfishness 2), four work ethic items (work ethics 1, 5, 6, 7), two appearance items (appearance 3, 4), and two items of human relations (human relations 4, 7).

We analyzed the measurement model using the CFA. The result from the CFA indicated that the measurement model included an adequate fit to the data (*χ*^2^ = 764.059, *df* = 362, *p* < 0.001, *χ*^2^/*df* = 2.111, RMSEA = 0.081, CFI = 0.882, IFI = 0.883, TLI = 0.867). Internal consistency for each research construct was evaluated. As shown in Table 3, results of the composite-reliability (CR) testing revealed that all reliability values were greater than Bagozzi and Yi’s [91] suggested cut-off of 0.600. The result proved the internal consistency of the measures for all variables used in this study. Consequently, construct validity was examined. As shown in Table 3. our calculation of the average variance extracted (AVE) generally supported the convergent validity as AVE of research constructs were close to or above the recommended value of 0.500 [92].

#### 4.3.2. Study 2: Confirmatory Factor Analysis (CFA)

Table 4 shows the confirmatory factor analysis. In general, the value of the standardization coefficient (λ) is judged to be statistically significant when it is greater than or equal to 0.5. Therefore, the item “That junior humiliated others in front of many people”, which has a standardization factor (λ) value of 0.479, has been deleted from the items corresponding to neuroticism.

As a result, 6 factors and 28 items—work ability (6), conscientiousness (3), selfishness (6), work ethics (3), appearance (2), neuroticism (8)—were finally derived.

As shown in Table 5, CFA result showed that the result of the measurement model included an adequate fit to the data (*χ*^2^ = 897.431, *df* = 362, *p* < 0.001, *χ*^2^/*df* = 2.479, RMSEA = 0.093, CFI = 0.901, IFI = 0.902, TLI = 0.889).

#### 4.3.3. Study 3: Measurement of Criterion Validity

In this study, Pearson’s correlation was used to analyze the correlation between variables. The correlation between all variables was found to be significant, and the contents are shown in Table 6. Looking at the inter-construction correlation coefficient, the correlation coefficient between work ability and job competency was the highest at 0.808. This was followed by selfishness and honesty (0.774), job competency and conscientiousness 2 (0.733), conscientiousness 1 and conscientiousness 2 (0.725), work ability and conscientiousness 1 (0.719), and work ability and conscientiousness 2 (0.715). Thus, all values were more than 0.7.

## 5. Conclusions

Table 7 shows the results of the stigma scale development for airline cabin crew. For the criterion validity analysis, a literature survey was conducted on the cause of stigma, and eight factors were identified. The eight factors are job competency, conscientiousness, honesty-humility, physical attractiveness, psychological well-being, mental health, leader member exchange (LMX), and perceived insider status (PIS). Based on this, the criterion validity was established. From the opinions of the first and second experts, who gave in-depth interviews, 7 factors and 29 items were derived. From these results, the exploratory factor analysis (EFA) resulted in a total of 28 items of six factors (work ability, conscientiousness, work ethics, appearance, selfishness, neuroticism).

**Figure 2:**
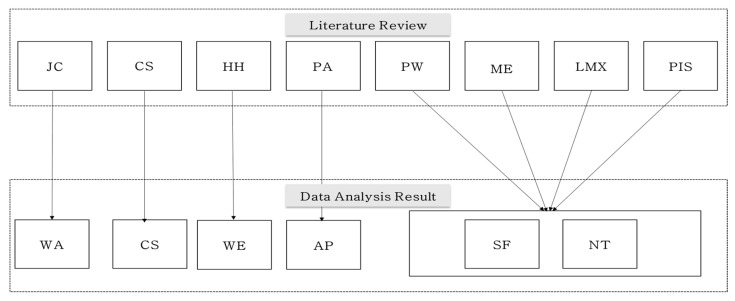
Result of scale development.

A total of 13 items that did not satisfy statistical significance were deleted from the initial items. As a result, the stigma scale developed for cabin crew consisted of a total of 6 factors and 28 items, including work ability (6), conscientiousness (3), selfishness (6), work ethics (3), appearance (2), and neuroticism. Figure 2. shows the results of the stigma scale development.

## 6. Discussion and Implications

Over the past few decades, American researchers have studied the stigma phenomenon of various social groups [93]. Many prior studies have studied the negative consequences associated with stigmatization. Some researchers have argued that stigma can be adjusted by informing people of potential risks and urging alternative actions [94,95].

The purpose of this study was to develop metrics for stigma-producing factors by conducting a survey among stigma inflictors, that is, people who participate in the stigmatization of others. The stigma scale for cabin crew members was composed of a total of six factors: work ability (6), conscientiousness (3), selfishness (6), work ethics (3), appearance (2), and neuroticism (8).

Work ability (WA) showed that cabin crew members often suffered alienation and stigmatization due to negative performance evaluations. Additionally, there were many cases where individuals who were unable to communicate well with other team members or did not understand the work properly, felt stigmatized.

Conscientiousness (CS) indicated that stigma occurs when a cabin crew member exhibits inadequacy at work, and neglects briefings or work. Due to the nature of the work that cabin crews do, team members are required to collaborate within a short period of time, and one of the team’s members may experience a setback in their work if they leave due to unfaithful factors.

Selfishness (SF) includes being obsessed with appreciation letters, being greedy, flattering and manipulating the leader, excessive devotion to the company to achieve success, and focusing solely on promotion.

Work ethics (WE) includes taking in-flight goods for personal use, lying at work, and the tendency to exaggerate (also described as mythomania depending on the situation). Work ethics as a moral and ethical factor refers to employees’ shared perception of what is right or wrong within the organization and how to deal with related ethical issues [96].

Appearance (AP) deals with violation of company attire regulations or the overall grooming being unsatisfactory. The appearance of the cabin crew represents not only an individual but also a company image, creating an overall image and atmosphere in an environment that provides services [97].

Lastly, neuroticism (NT) includes emotional ups and downs, violence in words and actions, hysteria, hypersensitivity, harassment of juniors, inclusion in the blacklist, causing disruption of teamwork, humiliating others in front of many people, and innocence. Some of these items were originally part of the human relations factor but were later integrated into neuroticism after looking at the statistical results of exploratory factor analysis.

The purpose of the study was as follows. First, this study attempted to develop a measurement scale for stigma factors by investigating the areas from which stigmas arise by looking through the perspective of stigma inflictors (senior cabin crew). These areas include work ability, conscientiousness, selfishness, appearance, work ethics, and neuroticism.

Second, this study attempted to develop a stigma scale for service industry workers, particularly airline cabin crew members. This study revealed how stigmatization occurs in the service industry and developed a scale for assessing stigma in the specific context of airline cabin crew members.

Third, by developing a stigma measurement scale, this study seeks to understand how stigma occurs among airline cabin crews in order to find solutions to minimize its negative effects. Stigma depends on social, economic, and political power, and has negative consequences for individuals and organizations, including loss of status, discrimination, stereotyping, alienation, and rejection [12]. By helping identify the causes of stigmas, the scale developed in this study can minimize such negative effects. The theoretical strength of this study is that the stigma measurement scale was developed from the perspective of the inflictors on the causes of workplace stigmatization among cabin crew members. Therefore, this study of stigmas is meaningful in that it was conducted on aviation industry workers who are the main pillars of the hospitality industry. The specific focus on cabin crew, the consultation with airline industry experts, and the participation of currently serving cabin crews differentiates the present study from existing stigma scale development studies.

The practical implications of this study are as follows. First, this study is meaningful as it analyses the causal factors of stigmas occurring within airline cabin crews. Second, by identifying the direct causes of workplace stigmas, they can be better prevented and self-stigmatization of victims can be minimized, thereby strengthening competency. Third, this study shows that all cabin crew members need to be faithful to their assigned responsibilities and roles in order to avoid being stigmatized. Fourth, for efficient performance at the organizational level, strategic measures should be taken to create a positive organizational culture such that stigmatization does not occur. Finally, victims who have suffered stigmatization must undergo psychological treatment to overcome the trauma of their stigma and improve their quality of life. At the organizational level, it should be emphasized that prevention is a more efficient way to manage airline personnel performance than post-trauma intervention.

## 7. Limitations and Future Research

First, stigma is a concept established by Goffman (1963), and various studies have been attempted, centering on the socially vulnerable classes. However, there is a relative lack of domestic and foreign literature on workplace stigmas. Notwithstanding the significance of developing a scale specifically for airline cabin crew, the present study is somewhat lacking in the literature review on the relationships within cabin crews and how stigmas affect them. In the future, we intend to conduct stigma research in various fields to create a foundation of research literature on workplace stigmas that could guide future researchers.

Second, this study conducted qualitative interviews and surveys only for cabin crews working for domestic South Korean airlines. Domestic cabin crews differ from international cabin crews in terms of working conditions and work environment. In future research, we will conduct a comparative analysis study on the causes of stigma not only in domestic cabin crews but also in the cabin crews of airline companies in other countries.

Third, the demographic analysis of this study showed the proportion of women to be significantly higher than that of men. Even though this reflects the general trend of more women being employed in airline cabin crews, it is somewhat regrettable that the gender ratio was not evenly distributed. In future studies, a survey will be conducted by subdividing the sample with a longer survey period. Finally, when a factor is defined by 4–5 items with loadings above 0.50, it is a solid factor with practical relevance but the factors conscientiousness (3), work ethics (3) and appearance (2) are defined by fewer items. The sample was not entirely representative of airline cabin crews. In future research, more participants will be interviewed to construct more items for each factor.

## Figures and Tables

**Table 1:** Comparison of initial items construct (literature studies vs. in-depth interviews)

Literature Studies	
**Job Competency** **(JC)**	
1	The junior lacked the ability to communicate effectively with passengers.
2	The junior lacked the ability to understand passengers’ needs.
3	The junior lacked the attitude to provide prompt service to passengers.
4	That junior lacked the ability to persuade passengers to accept the services or advice they were offering.
5	The junior lacked the professional ability to handle customer needs.
6	The junior lacked the knowledge to help passengers with service.
**Conscientiousness 1 (CS 1)**	
1	The junior doesn’t prepare her work in advance, and does it hastily at the last minute.
2	The junior sometimes had difficulties in working because she was not organized
3	The junior tends to do the least he needs.
4	The junior doesn’t care much about small details at work.
5	The junior didn’t always take the time to get things right.
6	The junior used to make impulsive rather than well-thought-out decisions.
7	He made a lot of mistakes because he didn’t think about it before acting.
**Honesty–Humility (HH)**	
1	The junior is someone who will use flattery or any other means to get a pay rise or a promotion.
2	That junior once laughed at someone’s worst joke to get something from them.
3	The junior had received a very large bribe.
4	The junior tends to get a lot of pleasure from owning luxury goods.
**Leader Member Exchange (LMX)**	
1	I don’t like the junior much.
2	I don’t want that junior to be my friend.
3	It is not pleasant to work with the junior.
4	When I was criticized by others, the junior didn’t stand by me.
5	The junior did nothing more than what was stated as his duties.
**Physical Attractiveness (PA)**	
1	The junior’s appearance is not very attractive.
2	The junior does not have a slim figure.
3	The junior is unkind to passengers.
4	The junior doesn’t care about his appearance and attire at all.
**Mental Health (MH)**	
1	The junior often looked nervous and anxious during briefings.
2	The junior was often less interested in briefing sessions.
3	The junior was often nervous and anxious about his in-flight work.
4	The junior was not interested in in-flight work.
5	The junior was often nervous and anxious during the layover.
6	The junior was often less interested in layover time.
7	The junior’s work did not show any potential of improving in the future.
**Psychological Well-Being (PW)**	
1	The junior did not seem to feel proud of his achievements.
2	The junior seemed to lack self-esteem and self-confidence.
3	The junior did not fulfill his daily responsibilities.
4	The junior did not seem to have a direction in his life.
5	The junior did not seem to take good care of his financial needs or personal affairs.
6	The junior did not seem to have someone who would listen to him in times of need.
**Perceived Insider Status (PIS)**	
1	The junior seemed to be “left-out” from the team.
2	The junior did not seem to get along well with his co-workers.
3	The junior’s presence in the team seemed to be irrelevant.
4	The junior did not seem to have sense of belonging with the team.
5	The junior seemed to be wandering on the periphery of the team.
**In-Depth Interviews**	
**Working Ability (WA)**	
1	The junior’s performance on the job was not good.
2	The junior did not achieve a good evaluation on the job.
3	The junior was often not familiar with their job description.
4	The junior did not receive a good assessment on their performance.
5	The junior had been disciplined.
6	The junior was unkind to the passengers.
7	The junior frequently received complaints from passengers.
8	There was a prejudice against the junior that he/she was incompetent.
9	The junior had difficulty communicating at work.
**Conscientiousness 2 (CS 2)**	
1	The junior was dishonest in his attitude to work.
2	The junior neglected his duties.
3	The junior was late for briefings.
**Selfishness (SF)**	
1	The junior was overly obsessed with getting a letter of appreciation.
2	There were rumors that the junior had a bad personality.
3	The junior was very greedy.
4	The junior had monopolized the boss to catch his boss’s eye.
5	The junior used a lot of flattery on his boss.
6	The junior had devoted himself blindly to the company to achieve success.
7	The junior was always preoccupied with the desire for promotion.
**Work Ethics (WE)**	
1	The junior did not obey business rules.
2	The junior had taken out the in-flight items.
3	The junior was prone to lying at work.
4	The junior has mythomania.
5	The junior had committed sexual harassment within the company.
6	The junior once stole other people’s belongings.
7	That junior had once made racist statements and committed racist actions.
**Appearance (AP)**	
1	I didn’t like the junior’s overall grooming.
2	The junior’s appearance did not comply with the regulations.
3	The junior didn’t smile much and didn’t look good.
4	The junior’s face had too much plastic surgery.
**Neuroticism (NT)**	
1	The junior had severe emotional ups and downs.
2	The junior was violent in his words and actions.
3	The junior was very hysterical at work.
4	The junior was easily upset or offended during conversations.
**Human Relations (HR)**	
1	The junior used his rank to harass subordinates.
2	The junior was on the company blacklist.
3	The junior broke his team work.
4	The junior had a bad relationship with his colleagues.
5	That junior humiliated others in front of many people.
6	The junior had no manners.
7	The junior’s personal life was unique and conspicuous.

**Table 2:** Exploratory factor analysis (EFA) and confirmatory factor analysis (CFA).

EFA							CFA		
Factor	Items	Com	FL	EV	VE (%)	α	λ	AVE	CR
WA (6)	W1	0.741	0.852	4.120	14.206	0.874	0.83	0.659	0.918
	W2	0.771	0.847				0.851		
	W3	0.752	0.851				0.802		
	W4	0.786	0.861				0.857		
	W5	0.459	0.569				0.561		
	W9	0.557	0.558				0.535		
CS (3)	C1	0.818	0.706	2.041	7.036	0.822	0.898	0.756	0.899
	C2	0.820	0.732				0.899		
	C3	0.788	0.704				0.554		
SF (6)	S1	0.712	0.813	4.448	15.337	0.916	0.742	0.605	0.901
	S3	0.613	0.674				0.667		
	S4	0.822	0.794				0.91		
	S5	0.760	0.796				0.871		
	S6	0.746	0.790				0.823		
	S7	0.780	0.851				0.808		
WE (3)	E2	0.681	0.653	2.517	8.679	0.804	0.674	0.743	0.895
	E3	0.837	0.842				0.892		
	E4	0.805	0.782				0.905		
AP (2)	A1	0.695	0.680	1.936	6.675	0.835	0.906	0.782	0.877
	A2	0.792	0.803				0.792		
N + H (9)	N1	0.676	0.778	5.799	19.998	0.925	0.848	0.617	0.866
	N2	0.716	0.793				0.783		
	N3	0.762	0.773				0.82		
	N4	0.801	0.856				0.778		
	H1	0.698	0.725				0.668		
	H2	0.633	0.675				0.700		
	H3	0.587	0.602				0.728		
	H5	0.549	0.629				0.758		
	H6	0.702	0.774				0.773		
KMO = 0.881, Bartlett *χ*^2^ = 3568.284, *df* = 406, *p* < 0.001									

Note: Com: Commonality, FL: Factor loadings, EV: Eigen values, AVE = Average variance extracted, CR: Composite reliability. WA = Work ability, CS1 = Conscientiousness 1, SS = Selfishness, WE = Work ethics, AP = Appearance, NT = Neuroticism, JC = Job competency, HH = Honesty–humility, CS2 = Conscientiousness 2, LMX = Leader member exchange, PA = Physical attractiveness, MH = Mental health, PW = Psychological well-being, PIS = Perceived insider status.

**Table 3:** Measurement model results (*n* = 170).

	1	2	3	4	5	6	AVE	CR
WA	1.000	0.526	−0.055	0.309	0.398	0.064	0.659	0.918
CS	0.277	1.000	0.228	0.49	0.488	0.497	0.756	0.899
SS	0.003	0.052	1.000	0.533	0.294	0.596	0.605	0.901
WE	0.095	0.240	0.284	1.000	0.411	0.485	0.743	0.895
AP	0.158	0.238	0.086	0.169	1.000	0.480	0.782	0.877
NT	0.004	0.247	0.355	0.235	0.230	1.000	0.617	0.866
*χ*^2^ = 764.059, *df* = 362, *χ*^2^/*df* = 2.111, GFI = 0.763, IFI = 0.883, TLI = 0.867, CFI = 0.882, RMSEA = 0.081								

Note: WA = Work ability, CS1 = Conscientiousness 1, SS = Selfishness, WE = Work ethics, AP = Appearance, NT = Neuroticism, JC = Job competency, HH = Honesty–humility, CS2 = Conscientiousness 2, LMX = Leader member exchange, PA = Physical attractiveness, MH = Mental health, PW = Psychological well-being, PIS = Perceived insider status

**Table 4:** Confirmatory factor analysis (CFA).

Factor	Items	λ	t-Value	AVE	C.R.	Factor	Items	λ	AVE	C.R.
WA (6)	W1	0.966		0.729	0.941	WE (3)	E2	0.739	0.788	0.842
	W2	0.97	34.658				E3	0.941		
	W3	0.908	24.167				E4	0.925		
	W4	0.946	29.477			AP (2)	A1	0.942	0.814	0.898
	W5	0.731	13.308				A2	0.939		
	W9	0.773	14.957			NT (9)	N1	0.769	0.637	0.939
CS1 (3)	C1	0.926		0.765	0.903		N2	0.845		
	C2	0.923	20.734				N3	0.684		
	C3	0.557	8.183				N4	0.763		
SF (6)	S1	0.727		0.624	0.908		H1	0.831		
	S3	0.728	9.547				H2	0.856		
	S4	0.932	12.399				H3	0.773		
	S5	0.917	12.188				H5	0.478		
	S6	0.827	10.921				H6	0.836		
	S7	0.884	11.727							

**Table 5:** Measurement model results (*n* = 170).

	1	2	3	4	5	6	AVE	C.R.
WA	1.000	0.907	0.260	0.798	0.914	0.809	0.729	0.941
CS1	0.823	1.000	0.246	0.804	0.885	0.783	0.765	0.903
SF	0.068	0.061	1.000	0.386	0.358	0.5	0.624	0.908
WE	0.637	0.646	0.149	1.000	0.78	0.757	0.788	0.842
AP	0.835	0.783	0.128	0.608	1.000	0.818	0.814	0.898
NT	0.654	0.613	0.250	0.573	0.669	1.000	0.637	0.939
*χ*^2^ = 897.431, *df* = 362, *χ*^2^/*df* = 2.479, NFI = 0.845, IFI = 0.902, TLI = 0.889, CFI = 0.901, RMSEA = 0.093								

**Table 6:** Result of Pearson correlation analysis.

	Mean	SD	1	2	3	4	5	6	7	8	9	10	11	12	13	14
WA	2.830	1.026	1	0.719 **	0.131 *	0.555 **	0.647 **	0.460 **	0.808 **	0.247 **	0.715 **	0.439 **	0.586 **	0.592 **	0.619 **	0.510 **
CS 1	2.588	1.060	0.719 **	1	0.249 **	0.579 **	0.654 **	0.575 **	0.660 **	0.366 **	0.725 **	0.478 **	0.604 **	0.601 **	0.615 **	0.465 **
SI	2.220	1.003	0.131 *	0.249 **	1	0.437 **	0.278 **	0.498 **	0.179 **	0.774 **	0.195 **	0.255 **	0.395 **	0.287 **	0.269 **	0.258 **
BE	2.341	1.090	0.555 **	0.579 **	0.437 **	1	0.578 **	0.578 **	0.570 **	0.472 **	0.584 **	0.351 **	0.620 **	0.539 **	0.493 **	0.400 **
AP	2.265	1.165	0.647 **	0.654 **	0.278 **	0.578 **	1	0.613 **	0.666 **	0.404 **	0.643 **	0.429 **	0.703 **	0.567 **	0.596 **	0.449 **
NP	2.370	0.976	0.460 **	0.575 **	0.498 **	0.578 **	0.613 **	1	0.516 **	0.579 **	0.502 **	0.543 **	0.688 **	0.586 **	0.568 **	0.479 **
JC	2.620	1.058	0.808 **	0.660 **	0.179 **	0.570 **	0.666 **	0.516 **	1	0.343 **	0.733 **	0.401 **	0.630 **	0.646 **	0.614 **	0.479 **
HN	2.031	0.906	0.247 **	0.366 **	0.774 **	0.472 **	0.404 **	0.579 **	0.343 **	1	0.350 **	0.348 **	0.511 **	0.420 **	0.405 **	0.360 **
CS 2	2.914	1.062	0.715 **	0.725 **	0.195 **	0.584 **	0.643 **	0.502 **	0.733 **	0.350 **	1	0.566 **	0.546 **	0.693 **	0.693 **	0.521 **
LMX	3.178	1.064	0.439 **	0.478 **	0.255 **	0.351 **	0.429 **	0.543 **	0.401 **	0.348 **	0.566 **	1	0.434 **	0.499 **	0.581 **	0.665 **
PA	2.270	1.014	0.586 **	0.604 **	0.395 **	0.620 **	0.703 **	0.688 **	0.630 **	0.511 **	0.546 **	0.434 **	1	0.595 **	0.636 **	0.521 **
MH	2.487	0.905	0.592 **	0.601 **	0.287 **	0.539 **	0.567 **	0.586 **	0.646 **	0.420 **	0.693 **	0.499 **	0.595 **	1	0.751 **	0.559 **
PW	2.517	0.889	0.619 **	0.615 **	0.269 **	0.493 **	0.596 **	0.568 **	0.614 **	0.405 **	0.693 **	0.581 **	0.636 **	0.751 **	1	0.667 **
PIS	2.565	0.957	0.510 **	0.465 **	0.258 **	0.400 **	0.449 **	0.479 **	0.479 **	0.360 **	0.521 **	0.665 **	0.521 **	0.559 **	0.667 **	1

Note: * Correlation is significant at the 0.05 level (2-tailed). ** Correlation is significant at the 0.01 level (2-tailed).

**Table 7:** Results of the stigma scale development for airline cabin crew.

Factor	Items	Results
WA (6)	The junior did not have a good performance on the job.	
	The junior did not receive a good evaluation on the job.	
	The junior was often not familiar with the job description.	
	The junior did not receive a good assessment on the job.	
	The junior had been disciplined.	
	The junior was unkind to the passengers.	Deleted
	The junior frequently received complaints from passengers.	Deleted
	There was a prejudice against the junior that he/she was incompetent.	Deleted
	The junior had difficulty communicating at work.	
CS2 (3)	The junior was dishonest in his attitude to work.	
	The junior neglected his duties.	
	The junior was late for briefings.	
SF (6)	The junior was overly obsessed with getting a letter of appreciation.	
	There were rumors that the junior had a bad personality.	Deleted
	The junior was very greedy.	
	The junior had monopolized the boss to catch his boss’s eye.	
	The junior used a lot of flattery on his boss.	
	The junior had devoted himself blindly to the company to achieve success.	
	The junior was always preoccupied with the desire for promotion.	
WE (3)	The junior did not obey business rules.	Deleted
	The junior had taken out the in-flight items.	
	The junior was prone to lying at work.	
	The junior has mythomania.	
	The junior had committed sexual harassment within the company.	Deleted
	The junior once stole other people’s belongings.	Deleted
	That junior had once made racist statements and committed racist actions.	Deleted
AP (2)	I didn’t like the junior’s overall grooming.	
	The junior’s appearance did not comply with the regulations.	
	The junior didn’t smile much and didn’t look good.	Deleted
	The junior’s face had too much plastic surgery.	Deleted
NT (8)	The junior had severe emotional ups and downs.	
	The junior was violent in his words and actions.	
	The junior was very hysterical at work.	
	The junior was easily upset or offended during conversations.	
	The junior used his rank to harass subordinates.	
	The junior was on the company blacklist.	
	The junior broke his team work.	
	The junior had a bad relationship with his colleagues.	Deleted
	That junior humiliated others in front of many people.	Deleted
	The junior had no manners.	
	The junior’s personal life was unique and conspicuous.	Deleted

## Data Availability

Data sharing is not applicable to this article.
